# Global initiative for childhood six-indexed cancers: how are we faring in Nigeria?

**DOI:** 10.3332/ecancer.2025.1993

**Published:** 2025-09-23

**Authors:** Motunrayo Oluwabukola Adekunle, Aisha Musa, Chioma Ginika, Chisom Nri-Ezedii, Uduak Offiong, Hauwa Yusuf, Peter Odion Ubuane, Adewunmi Oyesakin, Ijeoma Nnenna Diaku-Akinwumi, Adaorah Onyiaorah

**Affiliations:** 1 Lagos State University Teaching Hospital, Ikeja, Lagos State, Nigeria; 2 Aminu Kano Teaching Hospital, Kano, Nigeria; 3 University of Port Harcourt, Rivers State, Nigeria; 4 Nnamdi Azikiwe University Teaching Hospital, Nnewi, Anambra, Nigeria; 5 University of Abuja Teaching Hospital, Gwagwalada, FCT, Abuja, Nigeria; 6 University Teaching Hospital, Maiduguri, Borno State, Nigeria; 7 National Hospital, Abuja, Nigeria; a https://orcid.org/0000-0003-2820-3948

**Keywords:** Global Initiative for Childhood Cancer, treatment abandonment, treatment-related-mortalities, Nigeria

## Abstract

**Background:**

WHO's Global Initiative for Childhood Cancer (GICC) aims to increase global survival of childhood cancers to 60% by the year 2030 with a focus on six index cancers. However, there is no nationally representative epidemiologic data on these index cancers in Nigeria.

**Aim:**

To describe the distribution, outcomes and determinants of GICC six-indexed cancer in Nigeria.

**Methodology:**

A multi-centre ambi-directional cohort study of children was done in children <19 years diagnosed with any of acute lymphoblastic leukaemia (ALL), Wilms tumour (WT), retinoblastoma (RB), Hodgkin lymphoma (HL), Burkitt lymphoma (BL) or low-grade glioma (LGG). Seven centres in the six geopolitical zones of the country participated. A 2-year study with 18 months of retrospective data collection (January 2022–June 2023) and follow up of subjects was done for 6 months (July–December 2023).

**Results:**

A total number of 213 subjects were enrolled and ALL (*n* = 72;33.8%), WT (*n* = 57; 26.8%), RB (*n* = 52; 24.4%), BL (*n* = 17; 8.0%), HL (*n* = 13; 6.1%) and LGG (*n* = 2; 0.9%) accounted for the disease pattern. Median age at diagnosis was 5 years (51.6%). Mortality rate was 32.4% and treatment abandonment occurred in 37.6% of subjects. Treatment-related mortalities (TRMs) were 37.7% with infection and haemorrhage the commonest specific causes of TRM (36.1% and 42.5%). Only 7/95 (7%) of subjects with WT and RB stage III and IV benefited from RT. The most common reasons for non-RT were lack of funds (29%), lack of access to RT (54%) and lack of physicians’ referral (11%). Only 10 (4.3%) of subjects were enrolled in a health insurance scheme. Independent risk factor for mortality was advanced disease stage (*p* = <0.001). Amongst the mortalities, 36% died within the first 3 months of diagnosis.

**Conclusion:**

Treatment abandonment, mortality and TRM are high in Nigeria. To attain the GICC goal, there is a need to educate physician on treatment protocol, ensure availability of supportive care, health insurance, RT and tackle late presentation.

## Introduction

Globally, about 400,000 cases of childhood and adolescent cancers are diagnosed yearly. Childhood cancers are on the increase globally, most especially in Africa, with projections that Africa will account for 50% of childhood cancer in the world by the year 2050. Nigeria is not left out in the upsurge in childhood cancer [1, 2].

Childhood cancer is the leading cause of death in children and adolescents globally [1]. Mortality in childhood cancer is high with a wide disparity between the high and low-income countries. Whilst an 80% survival rate occurs in the former, the mortality rate in Africa is as high as 80% [1]. To close this gap, the global initiative for childhood cancer was initiated by WHO in the year 2018 with the aim to increase survival of childhood cancer to 60% by the year 2030 [3].

The six core malignancies of focus are acute lymphoblastic leukaemia (ALL), Nephroblastoma, Hodgkin lymphoma, retinoblastoma, Burkitt lymphoma and low-grade glioma. These malignancies account for 50%–60% of cancers, have good prognosis and are highly curable. Some of the measures towards attaining the goal of GICC by the year 2030 include prompt diagnosis, availability of essential medicines, availability of cancer registries and advocacy [3, 4].

Nigeria’s population is 223.8 million, with 43% of this accounting for children below 15 years [5]. There is paucity of data on childhood cancer in Nigeria. The vast majority of problems in care of childhood cancer span from lack of supportive care to unavailability of tools for molecular diagnosis [6]. The contributory effect of improvement of outcome in children with cancer in Nigeria to the achievement of GICC year 2030 is crucial.

This study aims to evaluate the six-core cancers of focus by GICC, their incidence, outcomes and determinants of the outcomes. The findings from this study will help to identify areas that require urgent attention and need to develop strategies that will enhance improvement of any of these malignancies lagging in contributing to the GICC goal 2030.

## Aim and objectives

The general aim of the study is to assess the incidence of six core malignancies in children in Nigeria, identify the outcomes, as well as factors that determine the outcomes.

The specific objectives of the study were to determine the incidence of ALL, Nephroblastoma, Hodgkin lymphoma, retinoblastoma, Burkitt lymphoma and low-grade glioma in study centres, the case fatality rates in the disease of interest over the study period as well as identify the contributor to mortality in the disease of interest over the duration of study (Age, sex, disease type, disease stage, time of presentation and treatment abandonment).

## Subjects and methods

**Figure 1.**
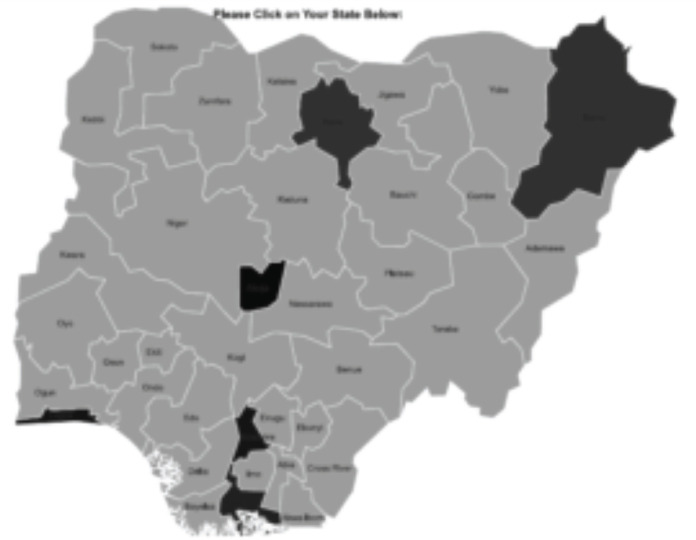
Map of Nigeria highlighting the study centres.

We conducted this multicentre collaborative national study at seven paediatric haemato-oncology units of seven teaching hospitals which provide treatment for children and adolescents with cancer across all six geopolitical zones of Nigeria (Figure 1):

1. Lagos State University Teaching Hospital, Ikeja, Lagos (South-West);
2. University of Port Harcourt, Rivers State (South-South);
3. Nnamdi Azikiwe Teaching Hospital, Nnewi, Anambra (South-East);
4. University of Abuja Teaching Hospital, Gwagwalada, FCT, Abuja (North-Central) and National Hospital, Abuja (North-Central);
5. University Teaching Hospital, Maiduguri, Borno State (North-East);
6. Aminu Kano Teaching Hospital, Kano (North-West).

### Study design

We employed an ambi-directional cohort design involving both a retrospective and prospective aspect. At each site, the site investigator used a standardised data collection form to extract relevant data of children below 19 years of age who were diagnosed and managed for any of the six GICC malignancies (acute lymphoblastic leukaemia, Hodgkin lymphoma, retinoblastoma, nephroblastoma, low grade glioma and Burkitt lymphoma) from January 1, 2022 till June 30, 2023 from hospital medical records. Enrolled patients who were still alive at the commencement of the study were thereafter prospectively followed up till December 31, 2023.

Data obtained include demographic (age and sex), clinical data (diagnosis and age at diagnosis), treatment resources (access to blood transfusion, radiotherapy, chemotherapy, surgery and supportive care) and outcome data (treatment abandonment, loss to follow-up, outcome at end of study – death versus survival). We defined *treatment-related mortality* as death occurring in the absence of disease progression [7]; *treatment abandonment* as failure to start or complete curative therapy (except when such treatment is contraindicated for medical reasons) and is defined by missed therapy for 4 or more consecutive weeks [8]; *curative treatment* is defined as treatment that is meant to cure the malignancy with the goal of a full recovery that includes an acceptable quality of life [9]. *Loss to follow-up* is defined as patients who have stopped follow-up appointment after completion of treatment for at least 3 consecutive months from the last appointment [10]; *events* are defined as any of death, relapse, progression, treatment abandonment and loss to follow-up. *Overall survival* is defined as the proportion of participants who were alive by the end of the study period [11]. All centres use International Society for Paediatric Oncology protocol for the treatment of Wilms tumour. A combination of vincristine, etoposide and carboplatin is used unanimously for the treatment of retinoblastoma. A combination of L-asparaginase, vincristine, 6-mecarpotopurine and prednisolone is used at different courses in the treatment of ALL. Five centres use cyclophosphamide, vincristine, Adriamycin and prednisolone in the treatment of BL, others use methotrexate-based regimen. A combination of Adriamycin, bleomycin, vinblastine and dacarbazine is the treatment protocol for HL in 5 centres, while 2 centres adopted cyclophosphamide, methotrexate, vincristine and prednisolone.

Ethical approval was obtained from the National Health Research Ethics Committee of Nigeria (NHREC) with approval number: NHREC/01/01/2007-12/10/2023.

### Data analysis

Data analysis was done using IBM SPSS Statistics version 20.0. Test of normality was assessed using Kolmogorov–Smirnov test. Patients’ demographics are represented as frequency and percentages. Tables and figures are used to represent those variables as appropriate. Continuous variables were summarised using mean and standard deviation, while non-parametric data were summarised using median. Comparison between parametric data was done using independent *t*-test and ANOVA test, while comparison of non-parametric data was done using Mann–Whitney test and Kruskal–Wallis. Comparison between qualitative data was done using chi square. Probability value less than 5% (0.05) was considered statistically significant. A survival analysis was done to determine the overall survival using Kaplan–Meier analysis.

## Results

A total of 213 patients were recruited with a slightly higher female proportion (0.9:1.0) in all the diseases. The median age at time of diagnosis is 5 years (IQR 11 months, 17 years). Adolescents aged 18 and 19 years were not seen in in the paediatric section of any institution. Patients with ALL, WT, RB and LGG presented at a significantly earlier years compared to those with BL and HL (χ^2^ 211.685; *p* = 0.020). The most are ALL (*n* = 72; 34%), WT (*n* = 57; 27%) and RB (*n* = 52; 24%). There was no case of bilateral Wilms tumour (stage V).

The median time to presentation to the hospital in all subjects from onset of symptoms is 90 days (IQR 6 days, 1095 days). The majority of the subjects presented with advanced disease. There was no relationship between disease stage and the type of malignancies (χ^2^ = 4.661, *p* = 0.550) as shown in Table 1.

**Figure 2.**
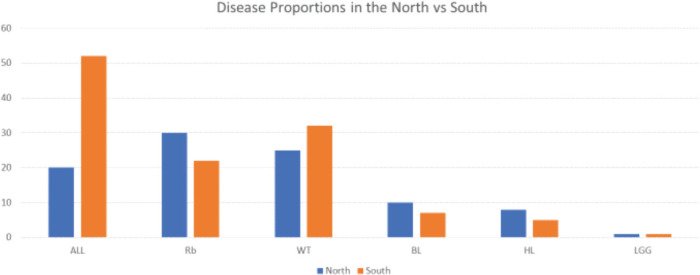
Proportion of diseases in the regions. ALL: acute lymphoblastic leukaemia; Rb: retinoblastoma; WT: Wilms tumour; BL: Burkitt lymphoma; HL: Hodgkin’s lymphoma; LGG: low grade glioma.

A higher proportion of ALL was diagnosed in the southern part of the country compared to the northern part (52/72; 72% versus 20/72; 28%,) while Burkitt lymphoma (10/17 (58.8% versus 7/17, 41.1%) and HL 8/13 (61.5^versus 5/13 (38.4%); were more common in the north than the south; χ^2^ 14.804, *p* = 0.010, as shown in Figure 2.

**Figure 3.**
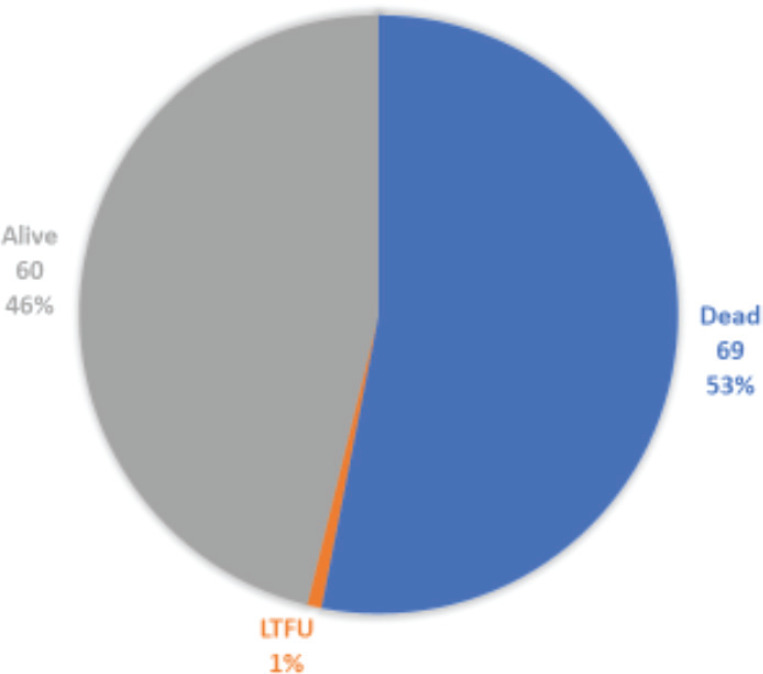
Disease outcomes in study subjects.

Out of 130 subjects, 53% died whilst 60 (46%) were still alive at the completion of the study, as shown in Figure 3. Early deaths within the first 3 months of diagnosis occurred in 25/69 (36%), and 44/66 (64%) deaths were recorded after the first 3 months of diagnosis.

Case fatality was highest in subjects with ALL while treatment abandonment was more common in those with WT and RB, as shown in Table 2.

**Figure 4.**
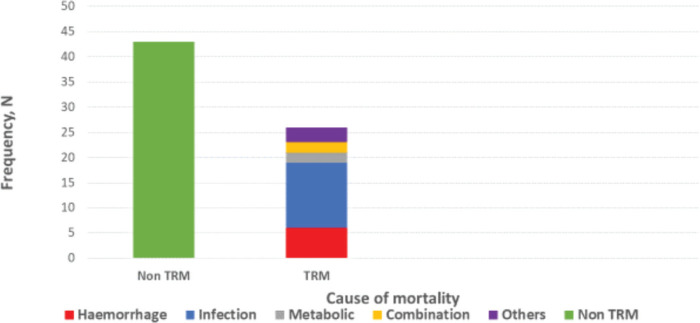
Causes of mortalities in study subjects.

Treatment-related mortalities occurred in 38% and the commonest cause is febrile neutropenia, as shown in Figure 4.

### Availability of services

**Figure 5.**
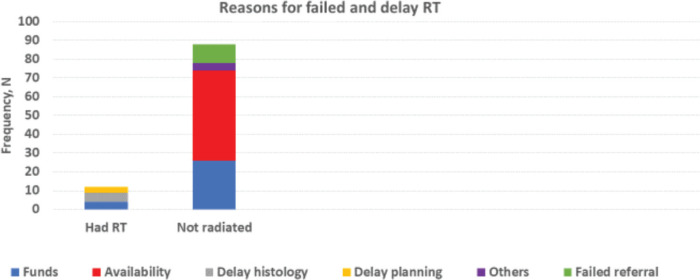
Radiotherapy services.

All centres have access to blood for transfusion. Four centres have equipment for pooled platelet and none for apheresis. Two centres have RT machines, while 1 has access to this service within the same city. All surgeons operate on patients; different treatment protocol exists in the study centres. A total of 109 patients had WT and RB; 95 of these required RT, i.e., WT (at least stage III) and RB (at least stage 1). Only 7 of these (7.4%) had the RT. None of the 5 patients with WT that had RT did so within 4 weeks post-surgery. No access to RT and delayed histology report were the commonest reasons for failure to irradiate when required and delayed irradiation, respectively, as shown in Figure 5.

### Predictors of treatment abandonment

Treatment abandonment was more common in the north than the southern part of the country, 41/92 (45%) versus 39/117 (33%) respectively (*p* = 0.065). No subjects on health insurance abandoned treatment and treatment abandonment occurred solely in those with no health insurance (*n* = 10, χ^2^ = 0.012, *p* = 0.008). Age and gender were not risk factors for abandoning treatment (*p* = 0.271 and 0.518 respectively). There is a 1.6 odd of not abandoning treatment in subjects with health insurance, as shown in Table 3.

Neither the distance from the facility of care to the home nor the access to health insurance were predictors of deaths. Subjects with advanced disease (WT, RB, HL and BL) had 23 odds of death compared to those with stages 1 and 2 disease at diagnosis, as shown in Table 4. Subjects with haematolymphoid diseases had a higher death rate compared to other malignancies. In all, 71% subjects with ALL died (*n* = 34/48) compared to 14/28 (50%) in retinoblastoma, 16/38 (42%) WT, 2/5 (40%) BL, 2/9 (22%) HL and one mortality in LGG (χ^2^ = 12.692, *p* = 0.020). Subjects with advanced diseases had a higher mortality rate than those with disease stages 1 or 2, as shown in Table 4.

## Discussion

To attain the goal of WHO GICC by the year 2030, there is a need for countries to evaluate where they are and strategically work towards the goal. The current study was done to access the current situation of children diagnosed with the six indexed malignancies and also evaluate factors that would hinder the required survival rate by the year 2030. The number of study participants in the current study is rather low despite being a multicentre study in major centres. The lack of national data on childhood cancer in Nigeria makes it difficult to extrapolate if this is as a result of preference for traditional care as against orthodox medicine or poor hospital data registry.

Acute lymphoblastic leukaemia is the commonest malignancy in our cohort with cancer. However, ALL tends to be commoner in the South while Burkitt lymphoma and Hodgkin’s lymphoma are commoner in the Northern part of Nigeria. The shift from HIV-related malignancies as the predominant malignancies has been previously documented and the pattern of disease now mirrors what obtains in HICs [12, 13]. The persistent high proportion of HIV related malignancies in the North has also been reported by Suleiman *et al* [14] The national shift to predominance of haematologic malignancies perhaps is as a result of clinical findings of a wide coverage of highly active antiretroviral therapy and winning in prevention of maternal-to-child transfusion of HIV. Likewise, the prevalence of low-grade glioma is low. This finding was also reported by Suleiman *et al* [14] The reduced immunity and increase malaria infection in individuals with low socioeconomic status and chronic malnutrition have been documented as a risk factors for BL; however, the evaluation of this parameters as contributor to regional disparities of BL is not within the scope of our study [15, 16]. The reason for the few brain tumours in our cohort is unclear. In a study done over a decade ago in an institution in the southwestern part of Nigeria, an approximately 0.76 cases per year low-grade astrocytoma was reported [17]. There is paucity of data on paediatric brain tumours in Nigeria, and this study further buttresses the need for research in this aspect of childhood cancer. In addition, multidisciplinary paediatric brain tumour clinics will enhance adequate capturing of cases.

In our cohort, the proportion of male subjects with BL and HL were remarkably higher than females. This is in keeping with the literature. The explanation for the increase risk of lymphoma in males is unknown with postulation of likely environmental or genetic factor [18, 19]. In addition, the proportion of female subjects with Wilms tumour in the present study was widely higher than males, this accounted for the overall gender distribution in our cohort. Girls have a higher risk of Wilms tumour than boys but the explanation for this risk is unclear [20].

The majority of our cohort presented late to the hospital and advanced disease stages were commonly found. This trend of delayed presentation has been reported in other LMICs; however, the time to presentation in our cohort is higher than 35 days reported by Ribeiro *et al* in Brazil and 68 days in Ethiopia [21, 22]. Children with retinoblastoma tend to present late. This was also reported by Gardie *et al* [22] in Egypt. The delayed presentation inadvertently resulted in more advanced diseases in study participants. As a country, there is a need to put measures in place to combat delayed diagnosis as this is crucial to GICC's goal achievement.

While it is acknowledged that high rates of both treatment abandonment and deaths are generally common in low-resource regions like Africa, the wide disparity in their prevalence amongst African countries cannot be ignored. A similar treatment abandonment rate was reported in an institutional-based study in the South-eastern part of Nigeria (51.2%), while 7% treatment abandonment was seen in a five-country study involving Ghana, Malawi, Cameroon, Zimbabwe and Kenya, and 42% in Ethiopia [23–25]. Studies have reported factors such as funding and preference for alternative medicine as a reason for treatment abandonment [24, 26]. As long as out-of-pocket financing of childhood cancer remains the norm in Nigeria, no significant impact can be made to reduce treatment abandonment. There is a need for the government to collaborate with international bodies on making treatment for childhood cancer free. There is a need for huge governmental and non-governmental investments to increase access to all cancer treatments and support, for example, through expansion of insurance coverage to the informal sector and for childhood cancer treatment. In addition, childhood cancer awareness programmes and education of the populace will enhance early presentation and improve survival outcome.

Our study reveals the potential positive impact of health insurance in that none of the few participants (< 5%) on health insurance abandoned treatment despite the fact that the scheme enrolled cancer patients, benefiting indirectly with respect to supportive investigations and non-oncologic medications. Childhood cancer treatment is yet to be included in the National Health Insurance Scheme in Nigeria. To attain the GICC goal, there is a need to expand the health insurance coverage beyond the current level of less than 5% enrolee and include childhood cancer treatment in the health insurance package [27].

The case fatality rate from our cohort was quite high compared to the other LMICs. In South Africa and Tanzania, Davidson *et al* [29] had a 77% survival rate and 18.9% deaths occurred in Tanzania, as reported by Majaliwa *et al* [28]. Subjects with ALL, Wilms tumour and retinoblastoma had the highest death ratio, 49%, 23% and 20%, respectively.

The high incidence of treatment-related mortality (death in the absence of progressive disease) in our cohort (38%), mostly due to febrile neutropenia and bleeding, is quite concerning, possibly reflecting weak resources for early detection and responses to such anticipated adverse events. This may also be due to the advanced disease at presentation, concomitant with a limited supportive care system. Along with enhanced supportive care system encompassing improved access to high-tech laboratory, molecular and blood product support, there is the additional need for evidence-informed adaptation of national treatment protocol with less treatment toxicity, contextualised to the peculiarity of late presentation in advanced disease stage when there is compromised reserve to withstand drug toxicities.

Although radiotherapy is a cornerstone treatment modality for WT, HL, RB and progressive LGG, the majority of the children who required radiotherapy could not access it (93%) due to unavailability of functional radiotherapy services, lack of funds, delayed histological results reporting and delayed clinical decision for radiotherapy. For Wilms tumour, radiotherapy within 4 weeks post-surgery has been documented to have a better prognosis, but none of our study participants commenced radiotherapy within a month after surgery. At a current estimated cost of 600 dollars per treatment, radiotherapy services may remain out of reach for a large proportion of children in a country where the average citizens earn 46 dollars. This further threatens the attainment of the GICC goal in Nigeria and further calls for concerted inter-sectorial interventions to subsidise radiotherapy services for childhood cancers. Additionally, timely cancer therapy hinges hugely on prompt histological diagnosis. Unfortunately, we observed significant delayed histological diagnosis, in addition to late presentation. Enormous manpower brain-drain in Nigeria’s health sector continues to worsen. There is thus a need for novel proactive measures to facilitate both histological investigations and reporting, for example, through AI.

Expectedly, children with advanced disease stage and haematolymphoid malignancies had increased odds of death, as similarly reported from both HIC and low- and middle-income countries. This partly explains our finding of low survival of 28%, a far cry from GICC goal of 60%! Worse still, these deaths occur mostly within 2 years post-diagnosis, similar to a study by Israels *et al* [30] who reported a mortality rate of 15% within the first 3 months among five sub-Saharan African countries (Nigeria not included). High rate of early deaths further strengthens the urgent need for strategies to enhance early detection and diagnosis and robust supportive care services.

## Strengths and limitation

Compared to several previously published single-centre cross-sectional or case-control studies, the multicentre ambi-directional cohort design of our study makes our study more representative of the current clinic-epidemiologic and resource challenge profile of paediatric oncologic services, specifically for GICC 6, in Nigeria. The combination of retro- and prospective aspects provided more data for better information. Conducting solely a retrospective study over a long period may be faced with the problem of data missingness, while a solely prospective study over a longer period may be limited by research resources. However, although all six Nigerian geopolitical zones were represented, we acknowledge that there are variations in the sociocultural contexts across each zone which may impact presentation, diagnosis and care.

In conclusion, ALL was the commonest of GICC-6. We report high incidence of treatment-abandonment, overall-mortality and TRM among Nigerian children with cancers; these factors threaten the attainment of GICC goal by Nigeria. There is thus an urgent need to enhance access/coverage of health-insurance for oncologic services, including radiotherapy, laboratory diagnostics and efficient referral pathways and linkages. Moreover, continuing multi-disciplinary and collaborative education of oncology care-providers in evidence-based locally-contextualised treatment protocols is required for optimal outcomes. Further prospective study to monitor progress and outcomes of advocacy should go side-by-side with these.

## List of abbreviations

ALL, Acute lymphoblastic leukaemia; BL, Burkitt lymphoma; GICC, Global Initiative for Childhood Cancer; HIC, High-income country; HL, Hodgkin’s lymphoma; LGG, Low-grade glioma; LMIC, Low-middle income country; RB, Retinoblastoma; RT, Radiotherapy; TRM, Treatment-related mortality; WHO, World Health Organisation; WT, Wilms tumour.

## Conflicts of interest

The authors hereby declare no conflicts of interest.

## Funding

There are no financial conflicts of interest to disclose.

## Figures and Tables

**Table 1.** Characteristics of the disease spectrum in the study participants.

Parameters	ALL *N* (%)	HL (%)	RB (%)	WT (%)	LGG	BL	Total (%)	Statistics
Gender Male Female Total	39 (38) 33 (30) 72 (34)	11 (11) 2 (2) 13 (6)	23 (22) 29 (26) 52 (24)	17 (16) 40 (36) 57 (27)	2 (2) 0 (0) 2 (1)	11 (11) 6 (5) 17 (8)	103 (100) 110 (100) 213 (100)	*χ*^2^ = 19.966 *p* = **0.001ǂ**
Median age at diagnosis (years) (IQR)	7 (5.11)	9 (7.12)	3 (2.4)	4 (2.8)	5 (4)	9 (7.12)	-	*χ*^2^ = 211.685 *p* = **0.004ǂ**
Time to presentation (days) (IQR)	60 (30.92)	90 (40.140)	225 (90.365)	90 (60.150)	120 (60)	51 (28.112)	-	*χ*^2^ = 212.161 *p* = < 0.001
Disease stage/severity 1 2 3 4 Unknown Total	NA	0 (0) 4 (15) 3 (8) 3 (5) 3 (25) 13 (9)	3 (43) 6 (23) 14 (36) 22 (40) 7 (58) 52 (37)	4 (57) 11 (43) 15 (38) 27 (49) 0 (0) 57 (41)	NA	0 (0) 5 (19) 7 (18) 3 (5) 2 (17) 17 (12)	7 (100) 21 (100) 32 (100) 52 (100) 12 (100) 139 (100)	*χ*^2^ = 10.227 *p* = 0.238

ǂ = significant value (*p* value <0.05)

ALL = Acute lymphoblastic leukaemia, HL = Hodgkin’s lymphoma; RB = retinoblastoma; WT = Wilms tumour; LGG = low grade glioma; BL = Burkitt lymphoma; UMTU = University of Maiduguri Teaching Hospital; AKTU = Aminu Kano Teaching Hospital; NAUTH = Nnamdi Azikiwe University Teaching Hospital; UPTH = University of Port Harcourt Teaching Hospital; LASUTH = Lagos State University Teaching Hospital; UATH – University of Abuja Teaching Hospital; IQR = interquartile range; NA = not applicable

**Table 2.** Treatment outcomes in study participants.

Treatment outcomes	ALL (%)	WT (%)	BL (%)	HL (%)	RB (%)	LGG (%)	Total (%)
Alive	14 (23)	22 (37)	3 (5)	7 (12)	14 (23)	0 (0)	60 (100)
Dead	34 (49)	16 (23)	2 (3)	2 (3)	14 (20)	1 (1)	69 (100)
Treatment abandonment	22 (27)	19 (24)	11 (14)	4 (5)	23 (29)	1 (1)	80 (100)
Transferred/LTFU	1 (25)	0 (0)	1 (25)	0 (0)	1 (25)	0 (0)	4 (100)
Total	73 (34)	57 (27)	17 (8)	13 (6)	52 (24)	2 (1)	213 (100)

*χ*^2^ = 28.622; *p* = 0.635

ALL= Acute lymphoblastic leukaemia, WT = Wilms tumour, BL = Burkitt lymphoma, HL = Hodgkin’s lymphoma,

RB = Retinoblastoma, LGG = Low grade glioma, LTFU = lost to follow-up

**Table 3.** Predictors of treatment abandonment.

Parameters	Abandoned treatment (%)	No treatment abandonment (%)	Total	*χ* ^2^	OR	*p* value
Age ≤5 years ≥6 years Total	41 (36) 39 (41) 80 (38)	73 (64) 56 (59) 129 (62)	114 (100) 95 (100) 209 (100)	0.568	0.806	0.271
Gender Male Female Total	39 (39) 41 (38) 80 (38)	62 (61) 67 (62) 129 (62)	101 (100) 108 (100) 209 (100)	0.009	1.028	0.518
Institution North South Total	41 (44) 39 (33) 80 (38)	51 (55) 78 (67) 129 (62)	92 (100) 117 (100) 209 (100)	2.750	1.608	0.065
Type of disease Haematolymphoid Solid & brain Total	38 (38) 42 (38) 80 (38)	62 (62) 67 (61) 129 (62)	100 (100) 109 (100) 209 (100)	0.006	0.978	0.525
Distance to hospital ≤100 km ˃100 km Total	29 (36) 45 (39) 74 (38)	51 (64) 70 (61) 121 (62)	80 (100) 115 (100) 194 (100)	0.166	0.885	0.399
Health insurance Yes No Total	0 (0) 80 (40) 80 (38)	10 (100) 119 (60) 129 (62)	10 (100) 199 (100) 209 (100)	6.513	1.672	**0.007ǂ**
*Duration of symptoms ≤30 days ˃30 days Total	11 (30) 57 (39) 68 (37)	25 (69) 89 (60) 114 (62)	36 (100) 148 (100) 184 (99)	0.889	0.687	0.228
**Disease stage 1–2 3–4 Total	8 (36) 29 (45) 37 (42)	14 (64) 36 (55) 59 (68)	22 (100) 65 (100) 87 (100)	1.665	0.527	0.147

*In 184 subjects with available information; ** In subjects with Wilms tumour, retinoblastoma, Burkitt’s, and Hodgkin’s lymphoma with confirmed disease staging

4 subjects transferred/lost to follow up excluded; OR = Odds ratio; km = kilometre

ǂ = significant value (*p* value <0.05)

**Table 4.** Predictors of death in study participants.

Parameters	Alive (%)	Dead (%)	Total	*χ* ^2^	OR	*p* value
Age ≤5 years ≥6 years Total	37 (62) 23 (38) 60 (100)	36 (52) 33 (48) 69 (100)	73 (100) 56 (100) 129 (100)	1.117	1.475	0.182
Gender Male Female Total	27 (45) 33 (55) 60 (100)	35 (51) 34 (49) 69 (100)	62 (48) 67 (52) 129 (100)	0.421	0.795	0.318
Institution North South Total	27 (45) 33 (55) 60 (100)	24 (35) 45 (65) 69 (100)	51 (40) 78 (60) 129 (100)	1.402	1.534	0.158
Type of disease Haematolymphoid Solid & brain Total	24 (40) 36 (60) 60 (100)	38 (61) 31 (46) 69 (100)	62 (100) 67 (100) 129 (100)	2.921	0.544	0.063
*Distance to hospital ≤100 km ˃100 km Total	25 (49) 34 (49) 59 (49)	26 (51) 36 (51) 62 (51)	51 (100) 70 (100) 121 (100)	0.002	1.018	0.554
Health insurance Yes No Total	5 (50) 55 (46) 60 (47)	5 (50) 64 (54) 69 (53)	10 (100) 119 (100) 129 (100)	0.053	1.164	0.537
**Duration of symptoms ≤30 days ˃30 days Total	10 (40) 41 (46) 51 (45)	15 (60) 48 (54) 63 (55)	25 (100) 89 (100) 114 (100)	0.291	0.780	0.380
***Disease stage 1–2 3–4 Total	17 (94) 18 (36) 35 (51)	1 ((6) 32 (64) 33 (49)	18 (100) 50 (100) 68 (100)	18.009	23.000	**<0.001ǂ**

*In 121 subjects with available information; **In 114 subjects with available information; *** In subjects with Wilms tumour, retinoblastoma, Burkitt and Hodgkin’s lymphoma with confirmed disease staging; ǂ = significant value (*p* value <0.05);

OR = odds ratio
